# Antimicrobial Properties of Silver-Modified Denture Base Resins

**DOI:** 10.3390/nano12142453

**Published:** 2022-07-18

**Authors:** Nikola Gligorijević, Tatjana Mihajlov-Krstev, Milena Kostić, Ljubiša Nikolić, Nemanja Stanković, Vesna Nikolić, Ana Dinić, Marko Igić, Nirit Bernstein

**Affiliations:** 1 Department of Prosthodontics, Faculty of Medicine, University of Niš, 18000 Niš, Serbia; milena.kostic@medfak.ni.ac.rs (M.K.); marko.igic@medfak.ni.ac.rs (M.I.); 2 Faculty of Sciences and Mathematics, University of Niš, 18000 Niš, Serbia; tatjanamk69@gmail.com; 3 Faculty of Technology, University of Niš, 16000 Leskovac, Serbia; nljubisa@tf.ni.ac.rs (L.N.); nikolicvesna@tf.ni.ac.rs (V.N.); anatacic@tf.ni.ac.rs (A.D.); 4 Institute for Public Health Niš, 18000 Niš, Serbia; nemanjastanko@yahoo.com; 5 Institute of Soil Water and Environmental Sciences, Volcani Center, Rishon LeZion 7505001, Israel; nirit@volcani.agri.gov.il

**Keywords:** denture base resins, silver nanoparticles, AgNPs, antimicrobial effect

## Abstract

The surface quality of denture base resins allows for easy colonization by microorganisms including *Candida albicans* and *Staphylococcus aureus*, which cause major diseases of the oral cavity such as denture stomatitis. The widespread use of silver nanoparticles (AgNPs) in various fields of medicine has led to research of their possible application in dentistry, mostly in the prevention of bacterial adhesion, proliferation, and biofilm formation. The aim of the study was to synthesize cold and heat-curing denture base resins modified with AgNPs and AgCl, and evaluate the potential of the modified resins to reduce the growth of *C. albicans* and *S.* *aureus*. The produced material was characterized by Fourier transform infrared spectroscopy (FTIR). The antimicrobial potential of the modified material was demonstrated by the disc-diffusion method, microdilution method, and a modified microdilution method (i.e., disk-diffusion method in broth with viable counting). Spectroscopy confirmed the incorporation of biocidal materials into the structure of the denture base resins. The AgCl and AgNPs modified resins showed an antimicrobial effect. The significance of the study is in the potential therapeutic effects of the modified materials for prevention and threating staphylococci and candida in elderly patients, who are in most cases denture wearers and have a greater susceptibility to develop opportunistic infections. Modified denture base resins can significantly reduce the presence of infection at the point of contact between the denture and the mucous membrane of the prosthetic restoration. Biological tests of modified denture base resins will follow.

## 1. Introduction

Given the growing number of toothless patients, the need to make mobile dentures is a medical and socio-economic challenge [1]. Due to their optimal mechanical and physicochemical properties and easy processing, dentures have been made for decades from heat-curing poly (methyl methacrylate) (PMMA) resins and repaired using cold-curing PMMA. However, the surface quality of acrylates allows easy colonization by microorganisms, including *Candida albicans*, *Streptococcus mutans*, *Staphylococcus aureus*, and other oral and non-oral microorganisms [2]. Biofilm formation on the surface of acrylic dentures can lead to a number of diseases of the oral cavity such as denture stomatitis, erythema, chronic mucositis, periodontitis and caries, as well as systemic diseases such as pneumonia and gastrointestinal disorders [3,4]. Studies have shown that denture stomatitis occurs in 20–70% of denture wearers, which reduces the value of the prosthetic restorations turning them into a collector of infectious material that can be disseminated throughout the oral cavity and the body [5]. Greater susceptibility for the development of oral infections in elderly denture-wearers patients is associated with a number of chronic diseases and decreased immunity, poor oral hygiene and less salivation, and there is a need to reduce the risk of infections with appropriate preventive methods [6,7]. Reducing the amount of biofilm on the surface of an acrylic denture would increase its biological value, and reduce the risk of oral infections and their systemic complications, and thereby also the need for expensive and often poorly controlled and recurrent therapy.

Numerous studies focused on the development of novel antimicrobial materials in order to overcome the problem of biofilm accumulation on oral tissue and materials implemented in the oral cavity. A main issue with the developed materials is their rapid degradation and thus inefficiency [8,9]. Therapy of denture stomatitis with antifungal drugs is mostly unsuccessful due to the multicausal etiology, resistance of microorganisms and insufficient concentration of the drug on the surface of the denture [10]. Denture cleansers are ineffective against typical denture plaque microorganisms, and also significantly damage acrylate dentures [11].

In recent years, nanoparticles (NPs) have emerged as important players in modern medicine [12], and are increasingly used and evaluated for their potential in imaging, diagnosis, drug delivery, tissue regeneration, and for the development of new medical material and products [13]. NPs are small solid particles with a diameter of 1–100 nm that can exhibit significantly different physical and chemical properties than their larger material counterparts [14]. Metal NPs were demonstrated to have a potential to be utilized as antimicrobial agents in a range of applications including medical instruments, food storage, wastewater treatment and dentistry [15,16,17,18,19,20,21,22]. Numerous NPs and NPs-based materials have been used as a new tool against microbial resistance and multidrug resistance [18]. After incorporation into a material, metal NPs have proven to be promising agents in antimicrobial therapy, which can be attributed to their physicochemical properties: ultra-small size, adequate surface-to-volume ratio and increased chemical reactivity [8,23,24].

Silver (Ag) has been well known for its antimicrobial characteristic and has a long history of application in medicine [25,26]. In dentistry it is used as an active compound against a broad spectrum of Gram-positive and Gram-negative bacteria [27,28,29,30,31] and fungi [32,33]. Silver ions (Ag^+^) bind to electron donor groups in biological molecules containing sulfur or nitrogen, resulting in defects in the bacteria cell membrane and leading to loss of their cell contents and to the death of bacteria [34]. Ag^+^ ions can interact with the DNA chain, preventing cell reproduction [34].

The widespread use of silver nanoparticles (AgNPs) in various fields of medicine has derived research towards their possible application in dentistry, mostly in the prevention of bacterial adhesion, proliferation and biofilm formation [35]. AgNPs in combination with polymers or as a smear or a liner on the surface of biomaterials show exceptional antimicrobial properties against oral microorganisms [36]. It therefore represents an alternative therapy that could be significantly used in clinical practice [37].

The aim of the study was to synthesize cold and heat-curing denture base resins modified with AgNPs and AgCl with the potential to reduce the growth of *Candida albicans* and *Staphylococcus aureus*.

## 2. Materials and Methods

### 2.1. Reagents

Silver nanoparticles-AgNPs (nanopowder, particles < 100 nm; Sigma Aldrich, Burlington, MA, USA), silver nitrate (Centrohem, Stara Pazova, Serbia), sodium chloride (Fisher Chemical, Loughborough, UK), potassium bromide (KBr, 99%, Merck, Darmstadt, Germany), methyl metacrylate-MMA and poly (methyl methacrylate)-PMMA (Triplex Hot and Triplex Cold, Ivoclar-Vivadent, Schaan, Lichtenstein).

### 2.2. Material Modification and Sample Production

Silver chloride (AgCl) was synthesized by dropping an aqueous solution of NaCl at a concentration of 1 mol/dm^3^ into an aqueous solution of AgNO_3_ of the same concentration with vigorous stirring (Homogenizer WiseTis HG-15A model, Witeg, Germany, 25,000 min^−1^) for 15 min. The resulting participate was filtered, washed with water and dried in a desiccator in the dark.

Modification of the material was performed by adding biocide powder to the concentrations of 2%, 5% and 10% for AgNPs and 10% for AgCl to the polymer powder, based on the total mass of the polymer-monomer mixture defined by the manufacturer’s instructions for both heat and cold-curing denture base resin (PMMA).

After the powder and monomer were mixed, the cold-curing dough was placed in a silicone disk-shaped molds 10 mm in diameter and 2 mm in height. Similarly, the heat-curing dough was placed in stone molds that had the same sample shape and size (10 mm diameter, 2 mm height) and put into standard denture flasks, pressed using a hydraulic press (Hydraulic dental press S-U-flask-press, Schuler-Dental GmbH & Co. KG, Ulm, Germany) (80 bar) and polymerized according to the manufacturer’s instructions. In short, closed flasks were put in cold water, heated up to 100 °C/212 °F and let to boil for 45 min. After the polymerization process, both the cold-curing and heat-curing samples were finished using dental acrylic laboratory burrs without polishing.

In this way 75 test samples per modification were produced, 750 test samples in total.Test specimens were labeled as 2%, 5% and 10% AgNPs PMMA heat and cold-curing, and 10% AgCl PMMA heat and cold-curing. Control samples were both cold and heat-curing PMMA without biocide agents (PMMA control heat and cold curing).

### 2.3. Fourier Transform Infrared Spectroscopy (FTIR)

The FTIR spectra of all examined samples and PMMA control samples were recorded using thin transparent pastilles made of 1 mg of tested samples and 150 mg of KBr (99%, Merck, Darmstadt, Germany) by vacuuming and pressing under the pressure of 200 MPa. The FTIR spectrum of the liquid monomer sample was recorded as a thin film between two zinc selenide (ZnSe) plates. FTIR spectra of all samples were recorded on a BOMEM MB-100 spectrophotometer (Hartmann & Braun, Frankfurt, Germany) in the wavenumbers range of 4000 to 400 cm^−1^ and analyzed using the Win-Bomem Easy software (ABB Bomen, Zurich, Switzerland).

FTIR analysis was performed for the PMMA samples containing 2%, 5%, 10% AgNPs, and for the PMMA samples containing 10% AgCl.

### 2.4. Scanning Electron Microscopy (SEM) Analysis

SEM analysis was used to examine the morphology of the AgNP/AgCl modified denture base resins. The samples were sprayed by an alloy of gold and palladium (85%/15%) under vacuum in a Fine Coat JEOL JFC-1100 Ion Sputter (JEOL Ltd., Tokyo, Japan). The metalized samples of the modified denture base resins were scanned using a JEOL SEM (JSM-5300, JEOL Ltd., Tokyo, Japan) at a voltage of 30 kV.

### 2.5. Test Organisms—Microbial Strains

Testing of the of modified denture base resins samples was performed against microbial strains that commonly grow in oral biofilm on the surface of dentures: the bacterial strain *Staphylococcus aureus* (ATCC 25923), and the yeast strain of *Candida albicans* (ATCC 1880). Suspensions were made from overnight cultures of microorganisms in sterile saline (0.9% NaCl), and turbidity was adjusted to 0.5 McFarland, corresponding to a cell number of 10^8^ CFU/mL. Antibacterial activity was tested by the disk-diffusion, microdilution and a modified microdilution method.

### 2.6. Disc Diffusion Antibiotic Sensitivity Method

Twenty mL of sterile Miller-Hinton agar (MHA, Oxoid LTD. Basingstoke, United Kingdom) or Saburodextrose agar (SDA, Liofilchem, Abruzzi (TE), Italy) were added to sterile petri dishes (90 mm in diameter) for the bacterial or the yeast strain antibiotic sensitivity test, respectively. After cooling, the plated media was inoculated with 100 µL of the prepared suspensions of the respective microbial strains, one strain per petri dish. After sterilization with a UV lamp, 3 discs per tested PMMA modification, 10 mm in diameter, were applied to each test sample. The petri dishes were then incubated at 37 °C for 18 h. After incubation, the diameter of the obtained zones of inhibition around each disk was measured. The analysis was performed with three replicates per each treatment.

### 2.7. Microdilution Method

The microdilution method was used for the determination of minimum inhibitory concentrations (MICs) and minimum microbicidal concentrations (MMC).

For the bacterial strain analysis, 100 µL of sterile Mueller-Hinton broth (MHB) was introduced into sterile 96-well microtiter plates with a dilution series of the modified denture base resin materials (10% AgNPs PMMA hot/cold curing, 10% AgCl PMMA hot/cold curing and PMMA control sample hot/cold-curing) in the concentration range of 0.02–50 mg/mL. The same procedure, as described above, was carried out for the analysis of yeast strains with the difference in the use 100 µL sterile Sabouraud Dextrose broth (SDB).

The medium was then inoculated with the appropriate suspensions so that the final volume in each well was 100 µL, and the final microbial cell concentration was 10^6^ CFU/mL.

Microtiter plates were incubated at 37 °C for 18 h. MICs were determined by monitoring visible colony growth following the addition of 0.5% triphenyltetrazolium chloride (TTC) which steins viable cells red. MMC were determined by sieving the contents of all wells in which there was no visible growth to sterile MHA or SDA in petri dishes. MMC is the concentration at which 99.9% of microbial cells are killed. The procedure was performed with three repetitions.

### 2.8. Modified Microdilution Method (a Broth Disk-Diffusion Method with Viable Counting)

The analysis was performed following Nam et al. [38] with some modifications. Sample disks of all tested materials and controls were placed in 22.1 mm diameter wells of 12-wells microtiter plates. 100 µL of the prepared microorganism suspension was applied to each disc. The microtiter plates were incubated at 37 °C for 90 min for the analysis of the direct contact action of the disc material on the microorganism cells. 1 mL of sterile broth (SDB for *C. albicans* and MHB for *S. aureus*) was then added to each well and the microtiter plates were incubated for 24 h at 37 °C. Determination of the degree of action of the disc shaped material samples on the tested microorganisms was performed by counting of viable microorganism cells. Namely, 100 µL of sample was taken from each well and a series of dilutions was made in sterile physiological solution. Then, 100 µL from dilutions 10^−2^, 10^−3^, 10^−4^, 10^−5^, 10^−6^ and 10^−7^ were inoculated on plate agar in Petri dishes (SDA for *C. albicans* and MHA for *S. aureus*). The plates were incubated for 24 h at 37 °C, the number of microbial colonies were counted and the standard number (number of colonies multiplied by dilution factor multiplied by correction to 1 mL) was calculated by the number of CFUs and the number of viable cells in 1 mL of the initial sample. The tests were performed with three independent replicates

### 2.9. Statistical Analysis

All measurements in the study were performed with three independent replicates. The data was analyzed by a one-way ANOVA, followed by Dunnett’s T3 post-hoc test for separation of means. The analysis was performed with SPSS software (ver. 15.0, Chicago, IL, USA). Statistical significance was defined as a *p* < 0.05.

## 3. Results

The starting components (MMA and PMMA cold and heat-curing), as well as 2%, 5% and 10% AgNPs PMMA and 10% AgCl PMMA (heat and cold-curing) were analyzed using the FTIR method.

**Figure 1:**
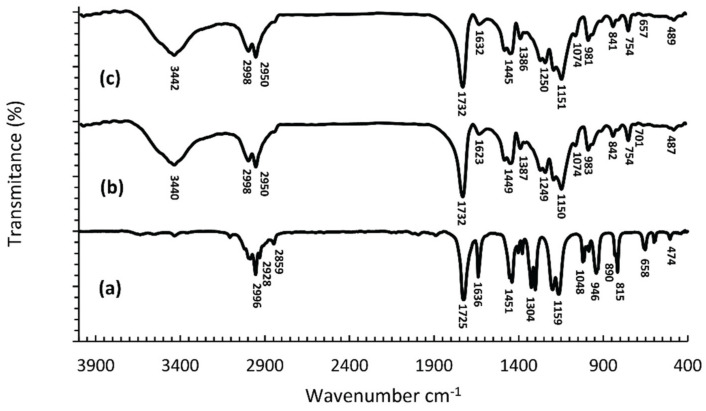
FTIR spectra of: MMA monomers (**a**), cold-curing PMMA polymers (**b**), and polymerized mixtures of monomers and cold-curing PMMA polymers (**c**).

**Figure 2:**
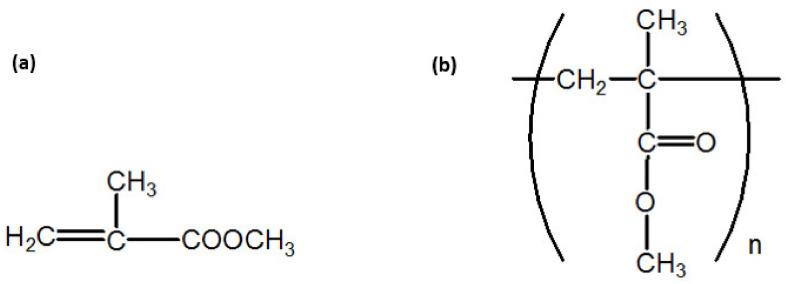
Structure of the MMA monomers (**a**) and the PMMA polymers (**b**).

Figure 1 shows the FTIR spectra of MMA monomers, cold-curing polymers and their polymerized mixtures, and Figure 2 presents the structures of the MMA monomers and PMMA polymers. In the spectra of the MMA monomers (Figure 1a), in the region of wave numbers above 2800 cm^−1^, three bands of low intensity can be observed at 2966 cm^−1^, 2928 cm^−1^ and 2859 cm^−1^. These bands originate from valence vibrations of the C-H bond from the -CH_3_ and the -CH_2_- groups. The prominent, sharp band at 1725 cm^−1^ is the result of valence vibrations of the C=O bond from the ester group, and was shifted to lower values of wave numbers, relative to its position in the spectrum of aliphatic esters (1750–1730 cm^−1^), due to the conjugation with the double bond. The band at 1636 cm^−1^ originates from the valence vibrations of the double bond. The valence vibrations of the C-O-C bond give two or more maxima in the wavelength range 1300–1000 cm^−1^ and in the MMA spectrum (Figure 1a) we observe two pronounced maxima at 1304 cm^−1^ and 1159 cm^−1^. Absorption bands at wavelengths less than 1000 cm^−1^ are attributed to different vibrations of C-H and C-C bonds [39].

In the spectrum of the synthesized PMMA (Figure 1b,c), in the range of wave numbers above 3000 cm^−1^ there is a wide band with a maximum at 3442 cm^−1^ corresponding to the valence vibrations of the OH group from adsorbed moisture [40]. Bands originating from valence vibrations of C-H bonds are of higher intensity and their maxima are shifted by 32 and 22 units, respectively, to higher values of wave numbers relative to the monomer spectrum, and occur at 2998 cm^−1^ and 2950 cm^−1^ [41]. The intensity of the band from the valence vibrations of the C=O group is unchanged, but its maximum has been shifted by 7 units to higher values of wave numbers, which together with the decrease of the band intensity from valence vibrations of the double bond and shifted its maximum by 4 units numbers shows that polymerization occurs by breaking double bonds [42]. Two bands of different intensities with maxima at 1449 cm^−1^ and 1386 cm^−1^ (in Figure 1b,c) originate from asymmetric and symmetric deformation vibrations, respectively, of CH_3_ groups. The valence vibrations of the C-O-C bond give two strong bands with maxima at 1250 cm^−1^ and 1151 cm^−1^ (Figure 1b,c), which is in agreement with the results of other authors [40]. The maxima at 981 cm^−1^, 841 cm^−1^ and 754 cm^−1^ originate from vibrations of C-H bonds outside the plane [43]. Differences in the appearance of FTIR spectra of monomers and polymers, i.e., differences in the position and intensity of characteristic maxima, primarily the loss of the band indicative of double bonding in the monomer, unequivocally confirm the synthesis of PMMA polymers (Figure 1). Comparative analysis of the FTIR spectra of PMMA polymers and cold and hot-curing PMMA (Figure 1b,c), show that they are identical, which is expected since they all represent polymerized MMA.

**Figure 3:**
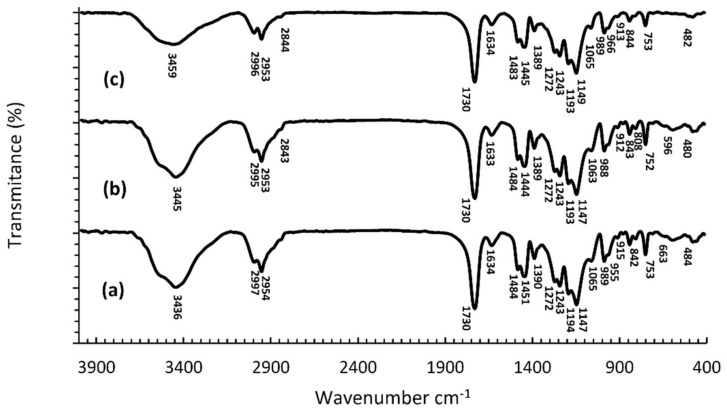
FTIR spectra of polymerized PMMA cold-curing (**a**), polymerized 10% AgNPs PMMA cold-curing (**b**), and (**c**) polymerized 10% AgCl PMMA cold-curing (**c**).

Figure 3 shows the FTIR spectra of synthesized cold-curing PMMA, for 10% AgNPs and 10% AgCl cold curing, respectively. The spectra with the highest content of biocidal agents are presented due to better detection of changes in the spectra. FTIR spectra of the cold-curing PMMA (Figure 3a) and the spectra of polymers with incorporated AgNPs/AgCl_3_ (Figure 3b,c), are similar in appearance. The biggest difference is reflected in the intensity and position of the maximum of the band originating from the valence vibrations of the OH group from adsorbed moisture, that its maximum was shifted by 9 and 23 units, respectively, towards higher values of wave numbers in the spectra with AgNPs or AgCl, respectively.

**Figure 4:**
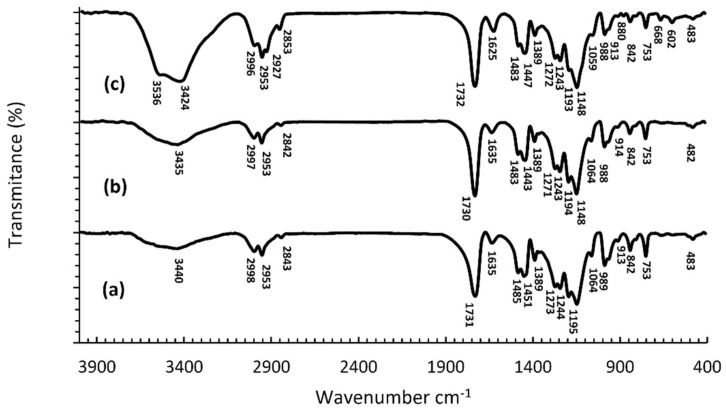
FTIR spectra of the polymerized heat-curing PMMA (**a**), the polymerized heat-curing 10% AgNPs PMMA (**b**), and the polymerized heat-curing 10% AgCl PMMA (**c**).

Figure 4a–c shows the FTIR spectra of the synthesized hot-curing PMMA, 10% AgNPs and the 10% AgCl heat-curing.

A comparative analysis of the FTIR spectra of heat-curing PMMA polymers (Figure 4a) with the spectra of the heat-curing PMMA polymers with incorporated AgNPs (Figure 4b,c) shows a considerable similarity in appearance. The most considerable difference between the materials is reflected in the intensity and position of the maximum of the band originating from the valence vibrations of the OH group from adsorbed moisture, whose intensity is higher, and the maximum is shifted by five units to lower values of wave numbers, in the spectrum with AgNPs.

The FTIR spectra of polymers with incorporated AgCl (Figure 4c) contains two new maxima, at 3536 cm^−1^ and 2927 cm^−1^, as well as an increase in intensity and shift of the maxima of bands present in the spectrum of the pure polymer (Figure 4a). Namely, the maximum of the band originating from the valence vibrations of the OH group from the adsorbed moisture was shifted by 16 units to lower values of wave numbers, while one of the maxima originating from the valence vibrations of the C-H bond from the -CH_3_ and -CH_2_- groups was shifted by 10 units towards higher values of wave numbers, and is located at 2853 cm^−1^ (Figure 4b,c). In the spectra of polymers with incorporated AgCl, an increase in the intensity of individual peaks, shift of the maximum, and even the appearance of new peaks at 3536 cm^−1^ and 2927 cm^−1^ was observed, which according to Shingho et al., 2012 [39] indicates the presence of silver chloride in the polymer.

**Figure 5:**
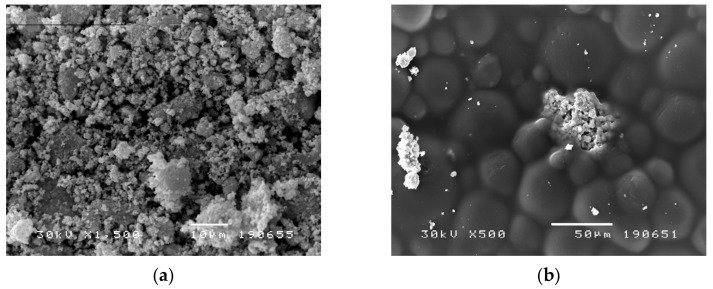
(**a**) SEM image of cold-curing PMMA with 10.0 wt% of AgNPs (1500×, bar 10 µm). (**b**) SEM image of cold-curing PMMA with 10.0 wt% of AgCl (500×, bar 10 µm).

Figure 5a,b represent backscatter electron images of cold-curing PMMA with 10 wt% AgNPs and 10 wt% AgCl. The SEM image (Figure 5a) confirms the presence of AgNPs in cold-curing modified PMMA, demonstrates their spread and distribution within the tested material. Figure 5b shows AgCl particle spread and distribution in the modified PMMA, but it is also noticeable that the homogenization of the AgCl particles in the polymer was not complete and thus the particles remained in associates. Also, it can be noticed that the sizes of individual AgCl particles is about 1 µm, which was achieved by the synthesis of AgCl.

Results of the microbiological tests performed are shown in Table 1 and Table 2. The results reveal an antimicrobial activity of 10% AgCl PMMA and 10% AgNPs PMMA for both cold and heat-curing samples (Table 1). In the case of lower concentrations of the biocidal substances, there was no diffusion into the substrate and consecutive inhibition of growth, but there was no microbial growth on the contact surfaces under the discs, which confirms their activity.

Minimum inhibitory concentrations (MIC) and minimum microbicidal concentrations (MMC) for the tested samples were determined by the microdilution method and the results are shown in Table 2. The best antimicrobial activity against *C. albicans* (MIC = MMC = 3.13 mg/mL) was identified for samples containing 10% AgCl PMMA, for both cold and heat curing PMMA (Table 2). Slightly lower activity of these samples was shown against *S. aureus* (MIC/MMC = 3.13/6.25 mg/mL). 10% AgNPs PMMA of both cold and heat-curing showed lower activity on the tested microorganisms compared to samples with the 10% AgCl PMMA, with MIC = MMC= 12.50 mg/mL (Table 2).

The modified microdilution method confirmed that under direct contact with the microorganisms all tested samples had a microbicidal effect (Table 3), with the exception of cold and hot-curing PMMA with 2% AgNPs that did not show an effect against *S. aureus*.

## 4. Discussion

In the present study we incorporated silver nanoparticles (AgNPs) and AgCl into the structure of the denture base reins polymer, confirmed the incorporation by SEM and FTIR analysis, demonstrated changes to the polymer spectra by the incorporation, and revealed the biocidal potential of the newly developed material.

AgNPs are considered promising agents in antimicrobial therapy due to their physicochemical properties: ultra-small size, large surface area, high charge density and increased chemical reactivity [8,24,44,45]. In contrast to antibiotics and antifungals, the use of which is limited by a number of contraindications and side effects as well as the limited duration of therapy, AgNPs in combination with polymers, smears or liners on the surface of biomaterials can provide a new strategy for treating and preventing oral infections [37,46]. The synergistic effect of AgNPs with conventional antimicrobial agents has been proven in previous studies [47,48]. High efficacy of AgNPs in combination with levoflorsacin was demonstrated against several Gram-positive and Gram-negative bacteria [49]; addition of AgNPs to biopolymers of carboxy methyl cellulose and sodium alginate proved their antibiofilms effect against Gram-positive and Gram-negative bacteria [50]; and other studies demonstrated high efficiency of AgNPs against Gram-negative bacteria [49,50]; anaerobic microorganisms [51]; excellent short/tearm and long-term antibacterial and biofilm ablation activity of AgNPs [52]; as well as novel use of Ag nanocatalysts in anti-infection therapy [53].

There is an increasing interest in the potential use of AgNPs in dentistry and promising recent information was accumulated recently, which served as the drive from the present study. Composite resins with AgNPs showed antibacterial activity against *St. mutans*, the cause of dental caries [54], long-lasting effect of low concentrations of AgNPs was demonstrated in adhesive materials [9], the combination of calcium phosphate AgNPs reduced metabolic activity of biofilm without reducing the binding strength of the applied adhesive [55], and addition of AgNPs to calcium disilicate cement increased its antimicrobial activity [56,57,58]. Furthermore, AgNPs were also shown to be effective against *E. faecalis* that is responsible for root canal reinfection [59,60,61,62], incorporation of AgNPs improved the therapeutic effect of Poly (vinyl alcohol) [63], Chlorhexidine-AgNPs inhibited *E. faecalis* [64], and the addition of AgNPS to periodontal dressing accelerated the healing of periodontal tissue [65]. Additional studies demonstrated that incorporation of AgNPs into the membrane for tissue-guided regeneration reduces bacterial adhesion [66], AgNPs coating of titanium implants prevents periimplantitis [67,68] as well as surface growth of *S. aureus* [68], and addition of AgNP to orthodontic hooks has a strong antimicrobial effect against *St. mutans* [69].

In the present study, SEM analysis of the tested materials confirms the presence of AgNPs and AgCl in both cold and heat-curing modified PMMA, demonstrates their spread, good dispersion and distribution within the tested material, which is in line with other authors finding in analogous research [70,71]. Comparative analysis of FTIR spectra of both heat and cold-curing PMMA revealed they are identical, which was expected since in both cases the material is a polymerized MMA. FTIR is a widespread analytical technique of chemical characterization [72]. The obtained results indicated high similarity of the obtained spectra for cold-curing synthesized PMMA and PMMA with incorporated AgNPs (i.e., incorporated AgCl at a concentration of 10%), as well as heat-curing PMMA with incorporated 10% AgNPs. On the other hand, the FTIR spectra of PMMA with incorporated 10% AgCl is characterized by the appearance of two new maxima, at 3536 cm^−1^ and 2927 cm^−1^, as well as an increase in intensity and shifts of the maximum bands present in the pure PMMA spectra. This indicates a possible easier oxidation on the surface of AgCl particles at elevated temperature to Ag_2_O, which creates conditions to participate in the formation of hydrogen bonds with polymethylmethacrylate segments. Elevated polymerization temperature also allows greater mobility of the segments of polymer chains and their more favorable orientation to form bonds with Ag_2_O. This creates the conditions to isolate new absorption bands in the FTIR spectrum that originate from O-H vibrations at 3536 cm^−1^ and valence C-H which participated in the formation of hydrogen bonds at 2927 cm^−1^. A FTIR absorption band derived from C-H valence vibration in the presence of silver at 2920 cm^−1^ has been shown before [73]. These processes are less favored for polymerization at lower temperatures, so these peaks in the FTIR spectrum do not occur in samples with 10% AgCl when polymerization is performed at room temperature. Possibilities of complexation and stronger interaction between these compounds have been published in the literature [74].

Similar reflection ranges of pure PMMA were detected by the FTIR—ATR method by Siddiqui et al. [75]. The spectra of pure PMMA and all nanocomposites were similar, indicating that the incorporation of AgNPs into the polymer matrix is mostly physical without a strong chemical bond. An analogous observation has been published in the literature [72,76]. The obtained results show that AgNPs PMMA nanocomposites resemble a solid solution with weak interaction between the polymer matrix and the nanoparticles.

The incorporation of NPs into denture base resins aims to avoid or at least reduce the colonization of dental materials by microorganisms, increasing the level of oral health and improving overall quality of life. Previous studies reported that the most common cause of denture stomatitis and stomatitis angularis is *C. albicans*, which is incorporated into the porous structure of denture base resins [77], although bacterial infection of *S. aureus* cannot be ignored [78]. In contrast to antibiotics and antifungal agents, whose use is limited by a number of contraindications and side effects, as well as by the limited duration of therapy, AgNPs may provide a new strategy for the treatment and prevention of oral infections in denture wearers [79]. The large surface area and high charge density of AgNPs imposes a significant impact on the negatively charged surface of bacterial cells resulting in enhanced antimicrobial activity [47]. In addition, a synergistic effect with conventional antimicrobials has been demonstrated [47,80].

The potential biocidal activity of modified acrylates is influenced by several factors: NPs size, solubility, shape, surface area and surface charge. These are the factors that determine both pharmacological activity and toxicity [81]. It is believed that smaller NPs are more efficient (i.e., that they release more ions in order to achieve optimal antibacterial effects). Lu et al. [51] showed that 5 nm AgNPs are most effective against anaerobic microorganisms. The use of Ag ions and salts as antimicrobial agents, whose positive charge is crucial for the antibacterial effect through electrostatic attraction with a negatively charged membrane, explains the antibacterial effects of the AgCl modified PMMA [82]. Their accumulation of Ag ions increases membrane permeability and causes cell death [28].

Accumulating information demonstrates that nanoparticles are able to bind and penetrate bacterial cell walls, and the silver ions can be released from them and alter cellular function [9,83]. The antibacterial effect was suggested to occur in three ways: interaction with the bacterial cell wall and the peptidoglycan membrane that causes cell lysis [9]; interaction with bacterial proteins and disruption of protein synthesis [84], and by interaction with bacterial (cytoplasmic) DNA and the prevention of DNA replication [85]. Silver is a safe antimicrobial agent that has the potential to kill 650 different types of disease-causing organisms [86], and AgNPs demonstrated antifungal activity against 44 strains of fungal species [32] and to inhibit respiratory chain enzymes and interfere with membrane permeability, which is attributed to the so-called “oligodynamic action” of Ag^+^ [87]. The most common modes of action of AgNPs are considered to be the prevention of DNA replication, formation of oxygen free radicals and direct damage to the cell membrane by silver ions [88], as well as denaturation and oxidize the cell wall leading to organelle rupture and cell lysis [28,89].

The results of the study showed antimicrobial activity of both cold and heat-cured 10% AgCl PMMA and 10% AgNPs PMMA. In the case of modification of denture base resins with lower concentrations of biocidal substances, their diffusion into the substrate and consecutive inhibition of growth did not occur, which can be explained by the size of the AgNPs (50–100 nm). On the other hand, there was no microbial growth on the contact surfaces of the substrate and the experimental sample discs (Table 1), which confirms their activity. Microbicidal effect in direct contact was also confirmed by a modified method with material discs in contact with viable cells in the broth (Table 3). Analogously, the modified denture base resins would be in direct contact with the mucous membrane, and their antimicrobial effect would be manifested even at lower concentrations of biocidal substances in the material. Clinically, the antimicrobial activity of dental materials by diffusion of antimicrobial elements is not necessary or desirable, since it depends on the flow of saliva and cannot be adequately controlled. On the other hand, there are opinions that long-term antimicrobial activity, with previously proven low toxicity and good biocompatibility in the human body, is the result of long-term release of ions and poorly developed resistance of bacteria to their action [90,91,92]. In this regard, the microdilution method results confirmed the microbicidal effect of the 10% AgNPs PMMA dilution against bacterial and fungal strains, while the effect of 10% AgCl PMMA dilution was somewhat weaker, especially against *S. aureus* strains.

Although membranes vary widely between mammals, bacteria, and fungi, they all contain phospholipids that contain negatively charged phosphate groups [93] incorporated between hydrophilic bases [93] which may be targets for membrane-distributed cationic AgNPs [94]. Lara et al. [95] have shown that the concentrations of AgNPs required for the inhibition of candida biofilm growth have a lower therapeutic potential than those shown by a standard cytotoxicity test on human hepatocellular carcinoma cell culture (HepG2). Panacek et al. [96] have shown that AgNPs in concentrations that have antifungal activity are not toxic to human fibroblasts. The advantage of AgNPs is their small size and the ability to penetrate cells, which increases their antimicrobial activity [97]. The results of some authors showed higher efficiency of AgNPs against Gram-negative bacteria [49,50]. The advantage of AgNPs is that they do not cause bacterial resistance, which complicates antibiotic therapy. Panacek et al. [96] compared and demonstrated the antifungal and antibacterial activities of AgNPs, and concluded that the effect on bacteria occurs under lower concentrations compared to fungi. Due to their less complex structure, evolutionarily older prokaryotic types of bacteria are unable to tolerate AgNPs as effectively as fungi that have a better organized structure and detoxification system [96].

Numerous studies reported that biofilms are significantly less susceptible to antimicrobial agents than planktonic cells [98,99,100]. Positively charged AgNPs not only inhibit the planktonic form of the fungus, but prevent the growth and consequent activity of the hyphal form in the in vitro culture model. Niska et al. [47] investigated the effect of AgNPs on bacteria isolates (Gram-positive and Gram-negative) isolated from patients with oral infections, as well as on commercial strains of *St. mutans* and *S. aureus*, proving their effectiveness.

## 5. Conclusions

The significance of the study is in the prevention and potential therapeutic effects of the modified materials in threating staphylococci and candida in elderly patients, who often wear dentures and are of greater susceptibility to developing opportunistic infections. Older age is associated with a higher prevalence of chronic diseases, immunodeficiency and malnutrition, as well as a decline in cognitive abilities and in some cases hygienic habits. FTIR analysis proved the incorporation of AgNPs and AgCl into the structure of the denture base reins polymer, and modified PMMA materials can significantly reduce the presence of infection at the point of contact between the denture and the denture support mucosa. Biological tests of modified denture base resins will follow.

## Figures and Tables

**Table 1:** Antimicrobial activity of samples against *S. aureues* and *C. albicans* strains tested by the disk-diffusion antibiotic sensitivity method. Zones of inhibition are expressed in mm. The results are averages ± S.E. (*n* = 3).

Sample	*S. aureus* ATCC 6538	*C. albicans* ATCC 24433
	*Inhibition zone* (mm)	
PMMA cold-curing	0.0000 ± 0.0000	0.0000 ± 0.0000
PMMA heat- curing	0.0000 ± 0.0000	0.0000 ± 0.0000
2% AgNPs PMMA cold-curing	0.0400 ± 0.0306	0.0200 ± 0.0058
2% AgNPs PMMA heat-curing	0.0400 ± 0.0306	0.0200 ± 0.0058
5% AgNPs PMMA cold-curing	0.0133 ± 0.0088	0.0300 ± 0.0058
5% AgNPs PMMA heat-curing	0.0077 ± 0.0062	0.0200 ± 0.0058
10% AgNPs PMMA cold-curing	9.0667 ± 0.0882 ***	9.0300 ± 0.1234 **
10% AgNPs PMMA heat-curing	9.0367 ± 0.0426 ***	9.0200 ± 0.0945 ***
10% AgCl PMMA cold-curing	9.0233 ± 0.0962 ***	9.0033 ± 0.2114 **
10% AgCl PMMA heat-curing	9.0033 ± 0.0561 ***	9.0667 ± 0.1348 ***
ANOVA (F, significance)	9046.24; *p* < 0.001	2486.63; *p* < 0.001

** *p* < 0.01, *** *p* < 0.001 based on Dunnett T3 Post-Hoc test (vs. both cold and heat-curing samples of PMMA and lower concentrations of AgNPs PMMA).

**Table 2:** Antimicrobial activity of samples against *S. aureus* and *C. albicans* strains tested by microdilution method (MIC/MMC). Minimum inhibitory concentrations (MIC) and minimum microbicidal concentrations (MMC) were determined with three independent repetitions.

Sample	*S. aureus* ATCC 6538	*C. albicans* ATCC 24433
	MIC/MMC, mg/mL	
10% AgNPs PMMA cold curing	12.50/12.50	12.50/12.50
10% AgNPs PMMA hot curing	12.50/12.50	12.50/12.50
10% AgCl PMMA cold curing	3.13/6.25	3.13/3.13
10% AgCl PMMA hot curing	3.13/6.25	3.13/3.13

**Table 3:** Antimicrobial activity of samples against *S. aureus* and *C. albicans* strains tested using by a modified microdilution method (a broth disk-diffusion method in broth with viable counting) (log-CFU/mL). The values are averages and SE (*n* = 3). Different letters near the means represent significant differences by Dunnett T3 test.

Sample	*S. aureus* ATCC 6538	*C. albicans* ATCC 24433
	Cell Count × 10^6^	
Growth Control	99.667 ± 0.882	99.667 ± 0.882
PMMA cold-curing	100.000 ± 0.000	101.000 ± 1.528
PMMA heat-curing	100.000 ± 0.000	100.667 ± 1.764
2% AgNPs PMMA cold-curing	100.000 ± 0.000	5.433 ± 0.260 a *** bc **
2% AgNPs PMMA heat-curing	100.000 ± 0.000	2.000 ± 0.012 a *** bc **
5% AgNPs PMMA cold-curing	0.133 ± 0.005 abc ***	0.001 ± 0.000 a *** bc **
5% AgNPs PMMA heat-curing	0.306 ± 0.005 abc ***	0.000 ± 0.000 a *** bc **
10% AgNPs PMMA cold-curing	0.000 ± 0.000 abc ***	0.000 ± 0.000 a *** bc **
10% AgNPs PMMA heat-curing	0.000 ± 0.000 abc ***	0.000 ± 0.000 a *** bc **
10% AgCl PMMA cold-curing	3.847 ± 0.144 abc ***	1.700 ± 0.115 a *** bc **
10% AgCl PMMA heat-curing	0.281 ± 0.012 abc ***	0.000 ± 0.000 a *** bc **
ANOVA (F. significance)	36,952.25; *p* < 0.001	3759.21; *p* < 0.001

**—*p* < 0.01, ***—*p* < 0.001 (Dunnett T3 Post Hoc test); (a—vs. Growth Control, b—vs. PMMA cold-curing, c—vs. PMMA heat-curing).

## Data Availability

The data presented in this study are available on request from the corresponding author.
